# Cr_5_B_3_ with the Shastry–Sutherland lattices

**DOI:** 10.1107/S2056989025006437

**Published:** 2025-07-29

**Authors:** Makoto Tokuda, Kunio Yubuta, Toetsu Shishido, Kazumasa Sugiyama

**Affiliations:** a Institute of Industrial Nano materials Kumamoto University, 2-39-1 Kurokami Chuo-ku Kumamoto 860-8555 Japan; b Institute for Aqua Regeneration, Shinshu University, 4-17-1 Wakasato, Nagano 380-8553, Japan; c Institute for Materials Research, Tohoku University, 2-1-1 Katahira, Aoba-ku, Sendai 980-8577, Japan; University of Hyogo, Japan

**Keywords:** single-crystal diffraction, crystal structure, boride

## Abstract

The structural parameters of penta­chromium triboride, Cr_5_B_3_, with Shastry–Sutherland lattices were refined based on single-crystal X-ray diffraction data. Cr_5_B_3_ crystallizes in the space group *I*4/*mcm* (No. 140), with the following lattice parameters: *a* = 5.4728 (1) and *c* = 10.0794 (2) Å. The present study succeeded in refining the positional and anisotropic atomic displacement parameters of the Cr and B atoms.

## Abstract 2

The structural parameters of penta­chromium triboride, Cr_5_B_3_, with Shastry–Sutherland lattices were refined based on single-crystal X-ray diffraction data. Cr_5_B_3_ crystallizes in the space group *I*4/*mcm* (No. 140). The present study succeeded in refining the positional and anisotropic atomic displacement parameters of the Cr and B atoms.

## 1. Chemical context

Several inter­metallic compounds, such as CrB_4_ (ortho­rhom­bic, *Immm*), CrB_2_ (hexa­gonal, *P*6/*mmm*), Cr_3_B_4_ (ortho­rhom­bic, *Immm*), CrB (ortho­rhom­bic, *Cmcm*), Cr_5_B_3_ (tetra­gonal, *I*4/*mcm*), *t*-Cr_2_B (tetra­gonal, *I*4/*mcm*) and *o*-Cr_2_B (ortho­rhom­bic, *Fddd*), exist in the binary Cr–B system (Lundström, 1969 ▸; Guy & Uraz, 1976 ▸; Massalski *et al.*, 2016 ▸). These binary chromium borides have attracted research attention as high-strength materials with excellent thermal, corrosion, and wear resistance. In addition to synthesis techniques (Okada *et al.*, 1996 ▸; Iizumi *et al.*, 1998 ▸), their applications in industrial fields have been also developed.

Notable magnetic (Guy, 1976 ▸; Leyarovska *et al.*, 1979 ▸), thermal (Leyarovska *et al.*, 1979 ▸) and transportation (Cruceanu *et al.*, 1975 ▸) properties have been reported in more than 130 inter­metallic compounds with a Cr_5_B_3_ prototype (ICSD, 2025 ▸). Anti­ferromagnetic ordering occurs in Cr_5_B_3_ at *T*_N_ = 91.5 K (Leyarovska *et al.*, 1979 ▸). The magnetic susceptibility and specific heat measurements of Cr_5_B_3_ suggest that its magnetic behavior originates from anti­ferromagnetic spin fluctuations in an itinerant electron system, rather than from localized magnetic moments (Leyarovska *et al.*, 1979 ▸). The observed effective magnetic moment of 0.02 *μ*_B_ per Cr atom indicates a low-spin state with significant *d*-electron delocalization. These findings imply that the formal oxidation state of Cr lies between +2 and +3. In the Cr_5_B_3_-type structure, metal atoms locate the two non-equivalent crystallographic sites. A two-dimensional square lattice formed by one of these metal sites can host magnetically frustrated orthogonal dimer systems. This square lattice is classified the Sharstry–Sutherland lattice (SSL; Shastry & Sutherland, 1981 ▸; Kageyama *et al.*, 1999 ▸; Siemensmeyer *et al.*, 2008 ▸; Coleman & Nevidomskyy, 2010 ▸). In particular, Ce_5_Si_3_, which has the Cr_5_B_3_-type structure, features a bilayer system of the SSL layers formed by Ce atoms, with additional inter­actions from out-of-plane Ce atoms contributing to its complex magnetic properties. (Ueta *et al.*, 2024 ▸).

The first structural investigation of Cr_5_B_3_ was conducted using a single crystalline sample (Bertaut & Blum, 1953 ▸). Both Cr and B atoms in the tetra­gonal Cr_5_B_3_ occupy two different Wyckoff sites, namely, 4*c* (0, 0, 0) and 16*l* (*x*, *x* + 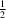, *z*) denoted as the Cr2 and Cr1 sites, respectively, and 4*a* (0, 0, 1/4) and 8*h* (*x*, *x* + 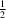, 0) denoted as the B1 and B2 sites, respectively. Subsequent structural studies on Cr_5_B_3_ have been limited to determining the lattice parameters, and the structural parameters have remained unchanged for over half a century (Portnoi *et al.*, 1969 ▸; von Robitsch, 1974 ▸; Paradelli & Gian­oglio, 1976 ▸; Hu *et al.*, 2014 ▸). Furthermore, regarding the atomic displacement parameters (ADPs) Bertaut & Blum (1953 ▸) did not determine them. Therefore, refining the anisotropic ADPs is essential for a more accurate structural description. Authors have reported that ADPs provide useful information about the structural properties of several metal boride compounds, such as YCrB_4_ (Tokuda *et al.*, 2022 ▸) and *RE*Rh_3_B_2_ (*RE* = Pr, Nd, and Sm) (Tokuda *et al.*, 2023 ▸). In this study, we update the structural parameters of Cr_5_B_3_ and perform a refinement, including the anisotropic ADPs.

Cr_5_B_3_-type (called T_2_-phase) analogous compounds have been studied using various experimental and theoretical methods, because they have many derivatives of the form *M*_5_*X*B_2_ (*M* = metal, *X* = non-metal or semi-metal such as Si, P, and Ge; Dahlqvist & Rosen, 2022 ▸). The structures of *M*_5_*X*B_2_ and Cr_5_B_3_ are related to the order–disorder atomic arrangements, in which *M*_5_*X*B_2_ and *X* atoms solely occupy the 4*a* site. Recently, several compounds of the form *M*_5_*X*B_2_ have been reported to exhibit superconductivity, such as Ta_5_GeB_2_(*T*_c_ ∼3.8 K; Hadi *et al.*, 2016 ▸; Corrêa *et al.*, 2016 ▸), Mo_5_GeB_2_ (*T*_c_ = 5.8 K; Ruan *et al.*, 2021 ▸), Mo_5_SiB_2_ (*T*_c_ = 5.8 K; Machado *et al.*, 2011 ▸), Mo_5_PB_2_ (*T*_c_ = 9 K; McGuire & Parker, 2016 ▸), and (W, Ta)_5_SiB_2_ (*T*_c_ = 6.5 K; Fukuma *et al.*, 2012 ▸).

## 2. Structural commentary

**Figure 1:**
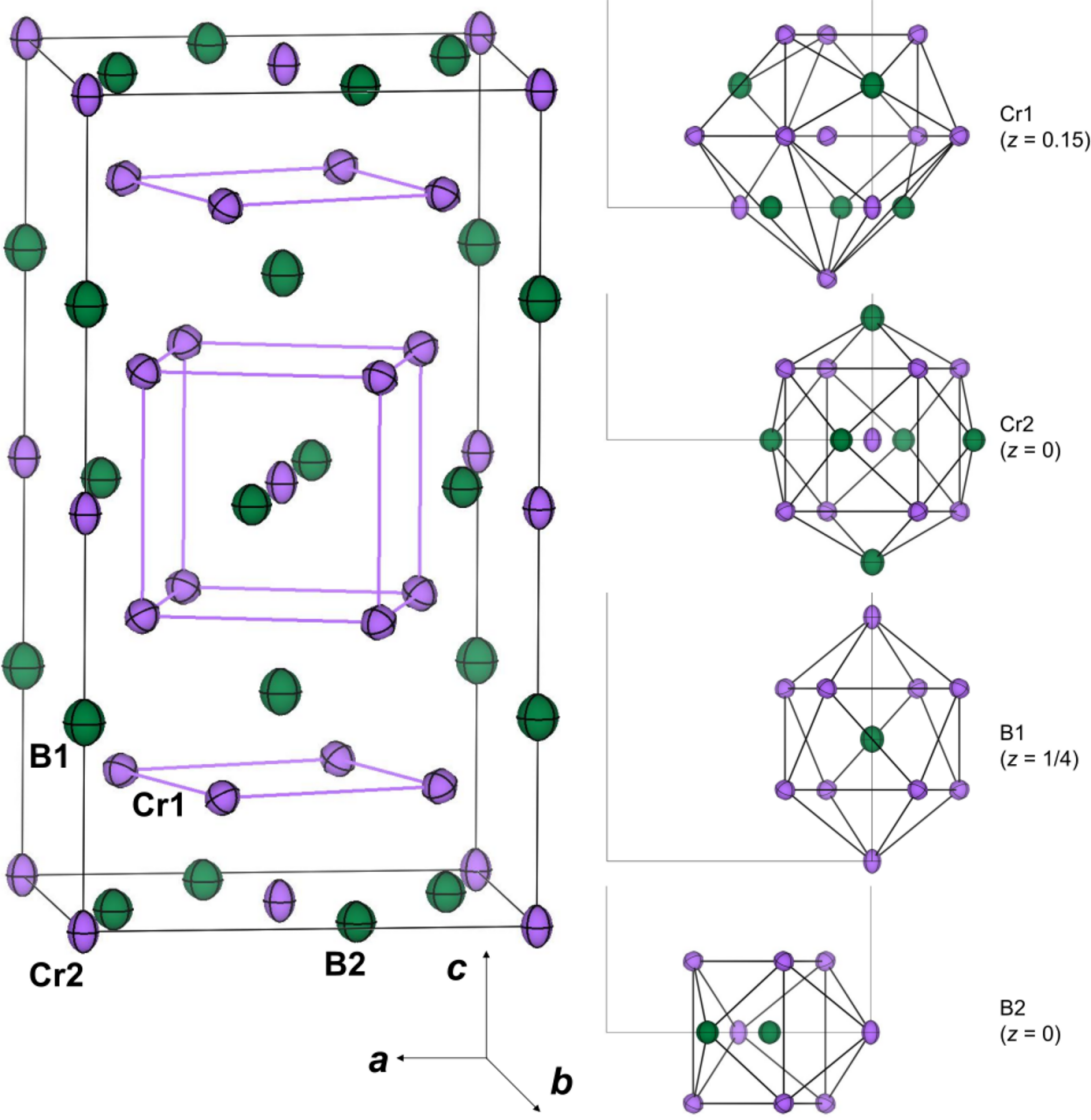
(left) Crystal structures of Cr_5_B_3_. The purple and green displacement ellipsoids correspond to Cr and B atoms, respectively. Displacement ellipsoids are drawn at the 99% probability level. (right) Coordination polyhedra for each site, Cr_11_B_5_ 16-vertex Frank–Kasper cluster around Cr1 on 16*l* site (*z* = 0.15), Cr_8_B_6_ rhombic dodeca­hedron around Cr2 on 4*c* site (*z* = 0), Cr_10_ bicapped square anti­prism around B1 on 4*a* site (*z* = 1/4), and Cr_8_B tricapped trigonal prism around B2 on 8*h* sites (*z* = 0).

In the Cr_5_B_3_ structure, the Cr atoms at the Cr2 and Cr1 sites are surrounded by a Cr_8_B_6_ rhombic dodeca­hedron and a Cr_11_B_5_ 16-vertex Frank–Kasper cluster, respectively, and those at the B1 and B2 sites are surrounded by a Cr_10_ bicapped square anti­prism and Cr_8_B tricapped trigonal prism, respectively (Fig. 1 ▸).

The B2—B2 inter­atomic distances on the *z* = 0 and 1/2 plane is 1.8168 (16) Å, which is significantly longer than the average B—B covalent bond distances of 1.77 Å in rhombohedral boron (Donohue, 1974 ▸). This B2 pair (B dimer) serves as a bridging unit between two adjacent Cr–B tricapped trigonal prisms, with each boron atom occupying the center of a respective polyhedron. The Cr—B inter­atomic distances are in the range of 2.1803 (3)–2.2826 (1) Å (Table 1 ▸), which are close to the sum of the Goldschmidt radii (*r*_Cr_ = 1.36 Å and *r*_B_ = 0.97 Å; Brandes & Brook, 1992 ▸). The intra­plane Cr2⋯Cr2 and intra­plane Cr2⋯Cr1 distances are 3.8698 (1) and 2.5072 (1) Å, respectively. The inter­plane Cr2⋯Cr1 distance is significantly smaller than the sum of the radii of the Cr atoms, and the anisotropic atomic displacement parameters (ADPs) for Cr2 exhibit a larger anisotropy than those of Cr1 (Table 2 ▸). The *U*_33_ of Cr2 is approximately 1.75 times larger than *U*_11_ (= *U*_22_), indicating that the displacement ellipsoid of Cr2 is elongated along the *c*-axis direction. These Cr⋯Cr distances and ADPs of Cr2 indicate a strong correlation between the Cr2 and Cr1 atoms.

**Figure 2:**
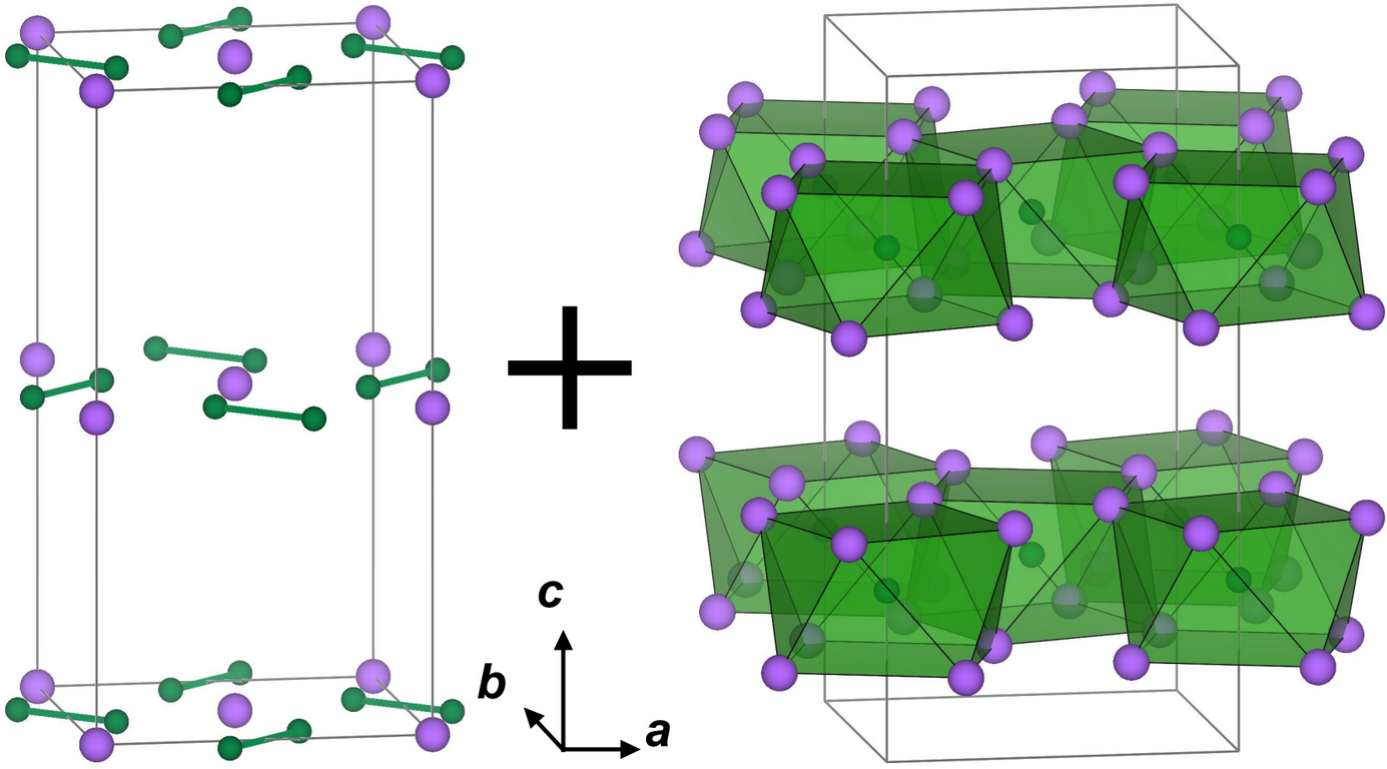
(left) Cr square lattices with B dimers at *z* = 0 and *z* = 1/2, and (right) slabs of Cr_8_B square anti­prisms around the B1 site between *z* = 0.15 and *z* = 0.35.

The bonding configurations of boron in metal boride compounds (*Mx*B*y*) are classified with the metal-to-boron ratio (*M*:B), and their structural characteristics have been systematically investigated (Rogl & Nowotny, 1978 ▸). In metal-rich compositions *M*/B > 1.5, boron typically exists as isolated B or a B–B dimer, occupying localized positions within the metal network. As the metal-to-boron ratio decreases, the bonding motifs transform progressively into mono-periodic chains (*e.g.*, Cr_2_AlB_2_-type), di-periodic boron layers (*e.g.*, CrB_2_-type), and eventually into three-dimensional frameworks constructed from B_6_ octa­hedra or B_12_ icosa­hedra (*e.g.* UB_4_-type). This structural diversity plays a crucial role in determining the electronic structure and physical properties of borides. In particular, Cr_5_B_3_, with a *M*:B of 5:3 (1.67), is categorized as a metal-rich boride and the boron configurations B1 and B2 sites correspond to ‘isolated B’ and ‘B–B dimer’, respectively. The slabs of Cr_8_ square anti­prisms [Cr⋯Cr inter­atomic distances of 2.42178 (14) and 2.86881 (3) Å] together with the B1 site and Cr square lattice [Cr⋯Cr inter­atomic distance of 2.50718 (5) Å] with B2 dimers [B2⋯B2 inter­atomic distance of 1.8168 (16) Å] alternately stack along the *c*-axis as shown in Fig. 2 ▸. The slabs of Cr_8_ square anti­prisms together with the B1 site connect via edge-sharing. Unlike boron-rich borides, the boron unit, Cr_5_B_3_, are locally confined, reflecting a characteristic inter­metallic bonding framework in which boron can act predominantly as an electron donor.

**Figure 3:**
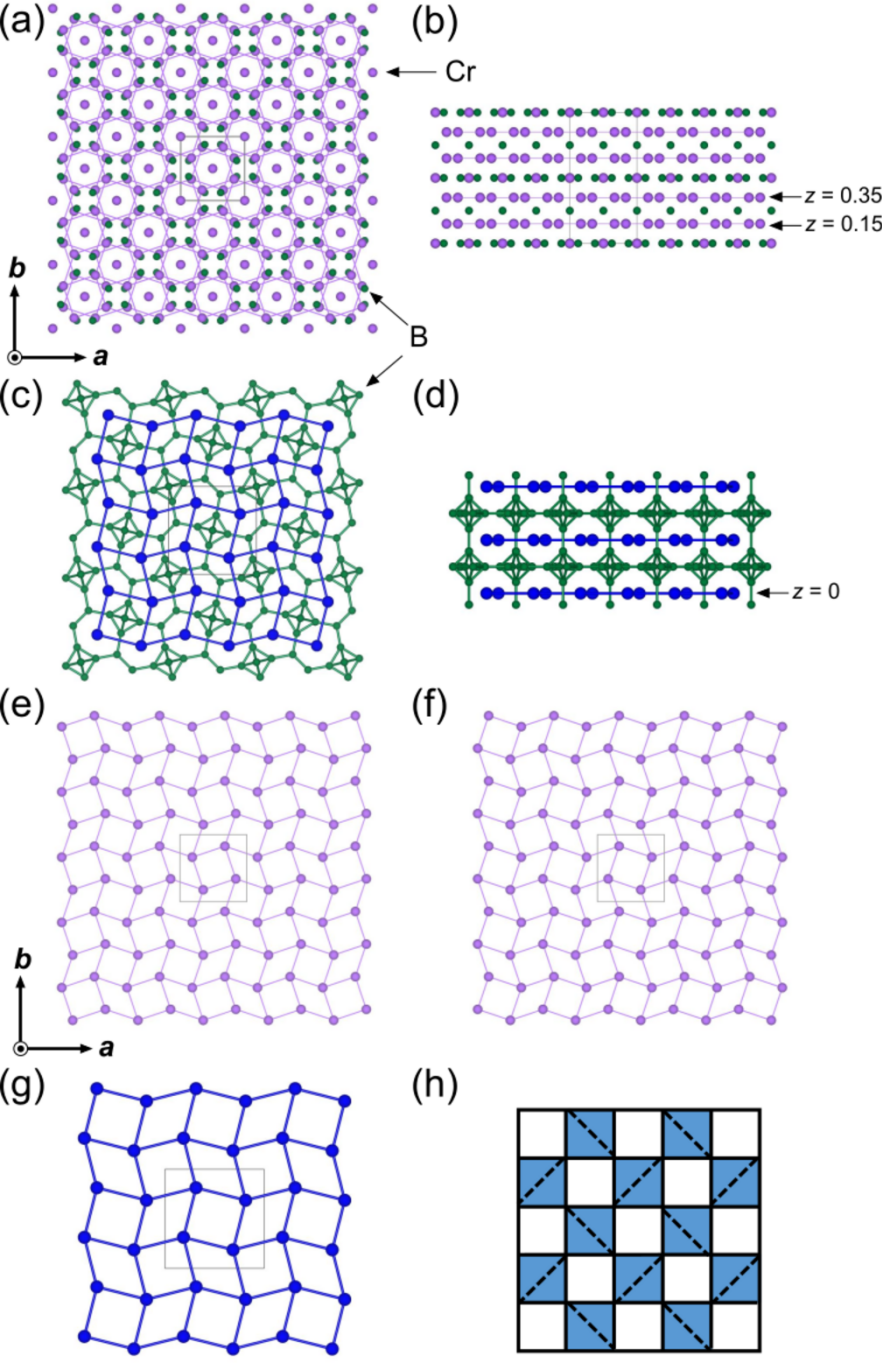
Crystal structures of binary metal borides: Cr_5_B_3_ (*a*, *b*) and TmB_4_ (*c*, *d*) viewed along the *c*-axis (*a*, *c*) and *b*-axis (*b*, *d*) directions. Red, green and blue spheres indicate Cr, B and Tm atoms. Gray squares in (*a*, *c*, *e*, *f*, *g*) are respective unit cells. Cr lattices at *z* = 0.15 (*e*) and *z* = 0.35 (*f*) in Cr_5_B_3_. (*g*) Tm lattice at *z* = 0 in TmB_4_. (*h*) Schematic of the SSL. The 1NN and the 2NN pairs are denoted by the solid and dashed lines, respectively.

Fig. 3 ▸ depicts the crystal structures of the binary metal borides Cr_5_B_3_ (*a*, *b*) and TmB_4_ (*c*, *d*) viewed along the *c*-axis (*a*, *c*) and *b*-axis (*b*, *d*). The purple, green and blue spheres indicate the Cr, B, and Tm atoms, respectively. The gray squares in (*a*, *c*, *e*, *f*, *g*) are the respective unit cells. Cr lattices [Cr⋯Cr inter­atomic distance of 2.6513 (1) and 2.8688 (1) Å] at *z* = 0.15 (Fig. 3 ▸*e*) and *z* = 0.35 (Fig. 3 ▸*f*) in the slabs of Cr_8_ square anti­prisms around B1 site of Cr_5_B_3_ (in the right panel of Fig. 2 ▸) are illustrated. The distribution of Cr are clearly demonstrated by square and triangle tilting. This tilting geometrical feature, composed of squares and triangles, is found in the TmB_4_ phase (Tm–Tm inter­atomic distances of 3.635 and 3.729 Å; Fisk *et al.*, 1972 ▸) (Fig. 3 ▸*g*) belonging to the UB_4_-type structure (tetra­gonal, *P*4*/mbm*). Both the Cr_5_B_3_ and TmB_4_ structures feature two-dimensional magnetic layers resembling the Shastry–Sutherland lattice (SSL), characterized by orthogonal dimers and inter­dimer inter­actions. However, while the SSL network in TmB_4_ is isolated within the structure, the SSL in Cr_5_B_3_ are embedded in a three-dimensional framework, making the magnetic frustration more complex and less idealized. This tiling structure is known as the SSL (Shastry & Sutherland, 1981 ▸). A schematic of the SSL is shown in Fig. 3 ▸*h*. The first-nearest-neighbor (1NN) and second-nearest-neighbor (2NN) pairs are denoted by the gray and black lines, respectively. Frustrated magnetic behavior with magnetic order is expected in Cr_5_B_3_-type analogous inter­metallic compounds consisting of SSL.

## 3. Synthesis and crystallization

Cr_5_B_3_ exhibits incongruent melting behavior during a peritectic reaction at 2247 K (Massalski *et al.*, 2016 ▸). Cr_5_B_3_ was obtained as a by-product of the synthesis of YCrB_4_ crystals. The starting materials were Y (99.9%), Cr (99.95%), and B (99.5%). The samples were weighed at an atomic ratio Y:Cr:B = 1:7:4. The mixtures were then melted in an Ar arc melting furnace (ACM-01, Diavac). The resulting button-like product was then turned over and remelted thrice to improve its chemical homogeneity. Single crystals for the X-ray diffraction measurements were obtained from the fractured surface of this button-like product.

## 4. Refinement

The refinement process was conducted for the space-group type *I*4*/mcm* as described by Bertaut & Blum 1953 ▸. A correction for isotropic extinction was applied during the least-squares refinement. The final refinements were performed by applying the anisotropic ADPs to each atom. These final refinement results are listed in Table 3 ▸. The refinement was successful, with the *R* factor converging without any problems and no noticeable residuals.

## Supplementary Material

Crystal structure: contains datablock(s) I. DOI: 10.1107/S2056989025006437/ox2016sup1.cif

Structure factors: contains datablock(s) I. DOI: 10.1107/S2056989025006437/ox2016Isup3.hkl

CCDC reference: 2473809

Additional supporting information:  crystallographic information; 3D view; checkCIF report

## Figures and Tables

**Table 1:** Selected bond lengths (Å) in Cr_5_B_3_

*Cr1:Cr_11_B_5_ 16-vertex Frank–Kasper*	
Cr1—B2×2	2.1803 (3)
Cr1—B2×1	2.2015 (6)
Cr1—B1×2	2.2826 (1)
Cr1—Cr2×1	2.4218 (4)
Cr1—Cr2×2	2.5072 (2)
Cr1—Cr1×1	2.6513 (3)
Cr1—Cr1×2	2.8098 (1)
Cr1—Cr1×4	2.8688 (1)
Cr1—Cr1×1	2.9468 (5)
	
*Cr2:Cr_8_B_6_ rhombic dodeca­hedron*	
Cr2—B2×4	2.1903 (4)
Cr2—Cr1×8	2.5072 (1)
Cr2—B1×2	2.5199 (1)
	
*B1:Cr_10_ bicapped square anti­prism*	
B1—Cr1×8	2.2826 (1)
B1—Cr2×2	2.5199 (1)
	
*B2:Cr_8_B tricapped trigonal prism*	
B2—B2×1	1.8168 (16)
B2—Cr1×4	2.1802 (3)
B2—Cr2×2	2.1903 (4)
B2—Cr1×2	2.2015 (6)

**Table 2:** Atomic coordinates and anisotropic atomic displacement parameters (10 ^3^ Å^2^) for Cr_5_B_3_ The Cr atoms lie on the Wyckoff site, the 4*c* site (0, 0, 0) and the 16*l* site (*x*, *x* + 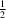, *z*), and the B atoms occupy the 4*a* site (0, 0, 1/4) and the 8*h* site (*x*, *x* + 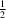, 0). The anisotropic displacement factor exponent takes the form 2π^2^[(ha*)^2^*U*_11_ + ⋯ + 2hka*b*U_12_]. *U*_eq_ is defined as a third of the trace of the orthogonalized *U*_*ij*_ tensor; *U*_11_ = *U*_22_, *U*_13_ = *U*_23_.

Atom	*x*	*z*	*U*_11_	*U*_33_	*U*_12_	*U*_13_	*U*_eq_
Cr1	0.17128 (2)	0.14618 (2)	0.00396 (2)	0.00369 (4)	−0.00001 (1)	−0.00022 (1)	0.00285 (3)
Cr2	0.0	0.0	0.00312 (6)	0.00547 (4)	0.0	0.0	0.00391 (2)
B1	0.0	0.25	0.00544 (17)	0.0066 (3)	0.0	0.0	0.00583 (12)
B2	0.38263 (10)	0.0	0.00489 (12)	0.0050 (4)	−0.00013 (15)	0.0	0.00492 (8)

**Table 3:** Experimental details

Crystal data	
Chemical formula	Cr_5_B_3_
*M*_r_	292.43
Crystal system, space group	Tetragonal, *I*4/*mcm*
Temperature (K)	294
*a*, *c* (Å)	5.47276 (5), 10.07939 (16)
*V* (Å^3^)	301.89 (1)
*Z*	4
Radiation type	Mo *K*α
μ (mm^−1^)	17.06
Crystal size (mm)	0.06 × 0.04 × 0.03

Data collection	
Diffractometer	XtaLAB Synergy, Dualflex, HyPix
Absorption correction	Gaussian (*CrysAlis PRO*; Rigaku OD, 2019 ▸)
*T*_min_, *T*_max_	0.520, 0.737
No. of measured, independent and observed [*I* > 2σ(*I*)] reflections	14650, 726, 687
*R*_int_	0.032
(sin θ/λ)_max_ (Å^−1^)	1.273

Refinement	
*R*[*F*^2^ > 2σ(*F*^2^)], *wR*(*F*^2^), *S*	0.011, 0.024, 1.10
No. of reflections	726
No. of parameters	16
Δρ_max_, Δρ_min_ (e Å^−3^)	0.61, −0.79

Computer programs: *CrysAlis PRO* (Rigaku OD, 2019 ▸), *SHELXT* (Sheldrick, 2015*a* ▸), *SHELXL2016/6* (Sheldrick, 2015*b* ▸) and *VESTA* (Momma & Izumi, 2011 ▸).

## supplementary crystallographic information

### Pentachromium triboride Crystal data

**Table:**

Cr_5_B_3_	*D*_x_ = 6.434 Mg m^−^^3^
*M**_r_* = 292.43	Mo *K*α radiation, λ = 0.71073 Å
Tetragonal, *I*4/*m**c**m*	Cell parameters from 8812 reflections
*a* = 5.47276 (5) Å	θ = 4.0–65.0°
*c* = 10.07939 (16) Å	µ = 17.06 mm^−^^1^
*V* = 301.89 (1) Å^3^	*T* = 294 K
*Z* = 4	Block, metallic
*F*(000) = 540	0.06 × 0.04 × 0.03 mm

### Pentachromium triboride Data collection

**Table:**

XtaLAB Synergy, Dualflex, HyPix diffractometer	726 independent reflections
Radiation source: micro-focus sealed X-ray tube, PhotonJet (Mo) X-ray Source	687 reflections with *I* > 2σ(*I*)
Mirror monochromator	*R*_int_ = 0.032
Detector resolution: 10.0000 pixels mm^-1^	θ_max_ = 64.8°, θ_min_ = 4.0°
ω scans	*h* = −13→12
Absorption correction: gaussian (CrysAlisPro; Rigaku OD, 2019)	*k* = −12→13
*T*_min_ = 0.520, *T*_max_ = 0.737	*l* = −25→25
14650 measured reflections	

### Pentachromium triboride Refinement

**Table:**

Refinement on *F*^2^	0 restraints
Least-squares matrix: full	*w* = 1/[σ^2^(*F*_o_^2^) + (0.0099*P*)^2^ + 0.1133*P*] where *P* = (*F*_o_^2^ + 2*F*_c_^2^)/3
*R*[*F*^2^ > 2σ(*F*^2^)] = 0.011	(Δ/σ)_max_ = 0.001
*wR*(*F*^2^) = 0.024	Δρ_max_ = 0.61 e Å^−^^3^
*S* = 1.10	Δρ_min_ = −0.79 e Å^−^^3^
726 reflections	Extinction correction: *SHELXL2016/6* (Sheldrick, 2015b), Fc^*^=kFc[1+0.001xFc^2^λ^3^/sin(2θ)]^-1/4^
16 parameters	Extinction coefficient: 0.0092 (5)

### Pentachromium triboride Special details

**Table:**

Geometry. All esds (except the esd in the dihedral angle between two l.s. planes) are estimated using the full covariance matrix. The cell esds are taken into account individually in the estimation of esds in distances, angles and torsion angles; correlations between esds in cell parameters are only used when they are defined by crystal symmetry. An approximate (isotropic) treatment of cell esds is used for estimating esds involving l.s. planes.

### Pentachromium triboride Fractional atomic coordinates and isotropic or equivalent isotropic displacement parameters (Å^2^)

**Table:**

	*x*	*y*	*z*	*U*_iso_*/*U*_eq_	
Cr01	0.17128 (2)	0.67128 (2)	0.14618 (2)	0.00387 (2)	
Cr02	0.000000	0.000000	0.000000	0.00391 (2)	
B01	0.000000	0.000000	0.250000	0.00583 (12)	
B02	0.38263 (10)	0.88263 (10)	0.000000	0.00492 (8)	

### Pentachromium triboride Atomic displacement parameters (Å^2^)

**Table:**

	*U*^11^	*U*^22^	*U*^33^	*U*^12^	*U*^13^	*U*^23^
Cr01	0.00396 (2)	0.00396 (2)	0.00369 (2)	−0.00001 (1)	−0.00022 (1)	−0.00022 (1)
Cr02	0.00312 (2)	0.00312 (2)	0.00547 (4)	0.000	0.000	0.000
B01	0.00544 (17)	0.00544 (17)	0.0066 (3)	0.000	0.000	0.000
B02	0.00489 (12)	0.00489 (12)	0.0050 (2)	−0.00013 (15)	0.000	0.000

### Pentachromium triboride Geometric parameters (Å, º)

**Table:**

Cr01—B02^i^	2.1803 (3)	Cr01—Cr01^viii^	2.8098 (1)
Cr01—B02^ii^	2.1803 (3)	Cr01—Cr01^ix^	2.8098 (1)
Cr01—B02	2.2015 (6)	Cr01—Cr01^x^	2.8688 (1)
Cr01—B01^iii^	2.2826 (1)	Cr02—B02^xi^	2.1903 (4)
Cr01—B01^iv^	2.2826 (1)	Cr02—B02^i^	2.1903 (4)
Cr01—Cr01^v^	2.4218 (1)	Cr02—B02^xii^	2.1903 (4)
Cr01—Cr02^vi^	2.5072 (1)	Cr02—B02^xiii^	2.1903 (4)
Cr01—Cr02^iv^	2.5072 (1)	B02—B02^xiv^	1.8168 (16)
Cr01—Cr01^vii^	2.6513 (1)		
			
B02^i^—Cr01—B02^ii^	49.25 (4)	Cr01^i^—Cr02—Cr01^xvi^	71.983 (3)
B02^i^—Cr01—B02	89.970 (13)	Cr01^vii^—Cr02—Cr01^xvi^	110.203 (2)
B02^ii^—Cr01—B02	89.970 (13)	Cr01^xv^—Cr02—Cr01^xvi^	69.797 (2)
B02^i^—Cr01—B01^iii^	96.830 (19)	Cr01^xiii^—Cr02—Cr01^xvi^	110.203 (2)
B02^ii^—Cr01—B01^iii^	145.619 (18)	B02^xi^—Cr02—Cr01^xii^	125.194 (13)
B02—Cr01—B01^iii^	96.230 (7)	B02^i^—Cr02—Cr01^xii^	54.806 (13)
B02^i^—Cr01—B01^iv^	145.619 (18)	B02^xii^—Cr02—Cr01^xii^	55.399 (13)
B02^ii^—Cr01—B01^iv^	96.830 (18)	B02^xiii^—Cr02—Cr01^xii^	124.601 (13)
B02—Cr01—B01^iv^	96.230 (7)	Cr01^xi^—Cr02—Cr01^xii^	69.797 (2)
B01^iii^—Cr01—B01^iv^	115.923 (3)	Cr01^i^—Cr02—Cr01^xii^	110.203 (2)
B02^i^—Cr01—Cr01^v^	152.867 (17)	Cr01^vii^—Cr02—Cr01^xii^	71.983 (3)
B02^ii^—Cr01—Cr01^v^	152.867 (17)	Cr01^xv^—Cr02—Cr01^xii^	108.017 (3)
B02—Cr01—Cr01^v^	101.803 (14)	Cr01^xiii^—Cr02—Cr01^xii^	180.0
B01^iii^—Cr01—Cr01^v^	57.961 (2)	Cr01^xvi^—Cr02—Cr01^xii^	69.797 (2)
B01^iv^—Cr01—Cr01^v^	57.961 (1)	B02^xi^—Cr02—Cr01^xvii^	55.399 (13)
B02^i^—Cr01—Cr02^vi^	55.184 (13)	B02^i^—Cr02—Cr01^xvii^	124.601 (13)
B02^ii^—Cr01—Cr02^vi^	94.138 (15)	B02^xii^—Cr02—Cr01^xvii^	54.806 (13)
B02—Cr01—Cr02^vi^	54.980 (5)	B02^xiii^—Cr02—Cr01^xvii^	125.194 (13)
B01^iii^—Cr01—Cr02^vi^	63.279 (1)	Cr01^xi^—Cr02—Cr01^xvii^	71.983 (3)
B01^iv^—Cr01—Cr02^vi^	149.178 (3)	Cr01^i^—Cr02—Cr01^xvii^	108.017 (3)
Cr01^v^—Cr01—Cr02^vi^	112.681 (3)	Cr01^vii^—Cr02—Cr01^xvii^	69.797 (2)
B02^i^—Cr01—Cr02^iv^	94.138 (15)	Cr01^xv^—Cr02—Cr01^xvii^	110.203 (2)
B02^ii^—Cr01—Cr02^iv^	55.184 (13)	Cr01^xiii^—Cr02—Cr01^xvii^	69.797 (2)
B02—Cr01—Cr02^iv^	54.980 (5)	Cr01^xvi^—Cr02—Cr01^xvii^	180.0
B01^iii^—Cr01—Cr02^iv^	149.178 (3)	Cr01^xii^—Cr02—Cr01^xvii^	110.203 (2)
B01^iv^—Cr01—Cr02^iv^	63.279 (1)	Cr01^xvii^—B01—Cr01^viii^	149.038 (3)
Cr01^v^—Cr01—Cr02^iv^	112.681 (3)	Cr01^xvii^—B01—Cr01^xiii^	77.867 (1)
Cr02^vi^—Cr01—Cr02^iv^	101.022 (3)	Cr01^viii^—B01—Cr01^xiii^	131.507 (3)
B02^i^—Cr01—Cr01^vii^	52.552 (7)	Cr01^xvii^—B01—Cr01^iii^	131.507 (3)
B02^ii^—Cr01—Cr01^vii^	52.552 (7)	Cr01^viii^—B01—Cr01^iii^	77.867 (2)
B02—Cr01—Cr01^vii^	137.988 (14)	Cr01^xiii^—B01—Cr01^iii^	64.078 (3)
B01^iii^—Cr01—Cr01^vii^	105.481 (2)	Cr01^xvii^—B01—Cr01^xviii^	64.078 (3)
B01^iv^—Cr01—Cr01^vii^	105.481 (2)	Cr01^viii^—B01—Cr01^xviii^	125.425 (3)
Cr01^v^—Cr01—Cr01^vii^	120.209 (3)	Cr01^xiii^—B01—Cr01^xviii^	75.975 (4)
Cr02^vi^—Cr01—Cr01^vii^	104.063 (2)	Cr01^iii^—B01—Cr01^xviii^	77.867 (1)
Cr02^iv^—Cr01—Cr01^vii^	104.063 (2)	Cr01^xvii^—B01—Cr01^i^	125.425 (3)
B02^i^—Cr01—Cr01^viii^	91.142 (10)	Cr01^viii^—B01—Cr01^i^	64.078 (3)
B02^ii^—Cr01—Cr01^viii^	114.398 (7)	Cr01^xiii^—B01—Cr01^i^	77.867 (2)
B02—Cr01—Cr01^viii^	148.111 (6)	Cr01^iii^—B01—Cr01^i^	75.975 (4)
B01^iii^—Cr01—Cr01^viii^	52.013 (2)	Cr01^xviii^—B01—Cr01^i^	149.038 (3)
B01^iv^—Cr01—Cr01^viii^	100.625 (3)	Cr01^xvii^—B01—Cr01^xix^	75.975 (4)
Cr01^v^—Cr01—Cr01^viii^	66.026 (4)	Cr01^viii^—B01—Cr01^xix^	77.867 (1)
Cr02^vi^—Cr01—Cr01^viii^	100.852 (2)	Cr01^xiii^—B01—Cr01^xix^	149.038 (3)
Cr02^iv^—Cr01—Cr01^viii^	156.417 (2)	Cr01^iii^—B01—Cr01^xix^	125.425 (3)
Cr01^vii^—Cr01—Cr01^viii^	61.849 (1)	Cr01^xviii^—B01—Cr01^xix^	77.867 (1)
B02^i^—Cr01—Cr01^ix^	114.398 (7)	Cr01^i^—B01—Cr01^xix^	131.507 (3)
B02^ii^—Cr01—Cr01^ix^	91.142 (10)	Cr01^xvii^—B01—Cr01^vii^	77.867 (2)
B02—Cr01—Cr01^ix^	148.111 (6)	Cr01^viii^—B01—Cr01^vii^	75.975 (4)
B01^iii^—Cr01—Cr01^ix^	100.625 (3)	Cr01^xiii^—B01—Cr01^vii^	125.425 (3)
B01^iv^—Cr01—Cr01^ix^	52.013 (2)	Cr01^iii^—B01—Cr01^vii^	149.038 (3)
Cr01^v^—Cr01—Cr01^ix^	66.026 (4)	Cr01^xviii^—B01—Cr01^vii^	131.507 (3)
Cr02^vi^—Cr01—Cr01^ix^	156.417 (2)	Cr01^i^—B01—Cr01^vii^	77.867 (2)
Cr02^iv^—Cr01—Cr01^ix^	100.852 (2)	Cr01^xix^—B01—Cr01^vii^	64.078 (3)
Cr01^vii^—Cr01—Cr01^ix^	61.849 (2)	Cr01^xvii^—B01—Cr02^xx^	117.287 (2)
Cr01^viii^—Cr01—Cr01^ix^	56.302 (3)	Cr01^viii^—B01—Cr02^xx^	62.713 (2)
B02^i^—Cr01—Cr01^x^	110.285 (13)	Cr01^xiii^—B01—Cr02^xx^	117.287 (2)
B02^ii^—Cr01—Cr01^x^	137.034 (5)	Cr01^iii^—B01—Cr02^xx^	62.713 (2)
B02—Cr01—Cr01^x^	48.782 (11)	Cr01^xviii^—B01—Cr02^xx^	62.713 (2)
B01^iii^—Cr01—Cr01^x^	51.067 (1)	Cr01^i^—B01—Cr02^xx^	117.287 (2)
B01^iv^—Cr01—Cr01^x^	98.917 (3)	Cr01^xix^—B01—Cr02^xx^	62.713 (2)
Cr01^v^—Cr01—Cr01^x^	63.500 (3)	Cr01^vii^—B01—Cr02^xx^	117.287 (2)
Cr02^vi^—Cr01—Cr01^x^	55.102 (1)	Cr01^xvii^—B01—Cr02	62.713 (2)
Cr02^iv^—Cr01—Cr01^x^	98.112 (3)	Cr01^viii^—B01—Cr02	117.287 (2)
Cr01^vii^—Cr01—Cr01^x^	152.478 (2)	Cr01^xiii^—B01—Cr02	62.713 (2)
Cr01^viii^—Cr01—Cr01^x^	101.560 (1)	Cr01^iii^—B01—Cr02	117.287 (2)
Cr01^ix^—Cr01—Cr01^x^	129.526 (3)	Cr01^xviii^—B01—Cr02	117.287 (2)
B02^xi^—Cr02—B02^i^	180.0	Cr01^i^—B01—Cr02	62.713 (2)
B02^xi^—Cr02—B02^xii^	90.0	Cr01^xix^—B01—Cr02	117.287 (2)
B02^i^—Cr02—B02^xii^	90.0	Cr01^vii^—B01—Cr02	62.713 (2)
B02^xi^—Cr02—B02^xiii^	90.0	Cr02^xx^—B01—Cr02	180.0
B02^i^—Cr02—B02^xiii^	90.0	B02^xiv^—B02—Cr01^x^	65.376 (19)
B02^xii^—Cr02—B02^xiii^	180.00 (3)	B02^xiv^—B02—Cr01^xxi^	65.376 (19)
B02^xi^—Cr02—Cr01^xi^	55.399 (13)	Cr01^x^—B02—Cr01^xxi^	74.894 (14)
B02^i^—Cr02—Cr01^xi^	124.601 (13)	B02^xiv^—B02—Cr01^xxii^	65.376 (19)
B02^xii^—Cr02—Cr01^xi^	54.806 (13)	Cr01^x^—B02—Cr01^xxii^	130.75 (4)
B02^xiii^—Cr02—Cr01^xi^	125.194 (13)	Cr01^xxi^—B02—Cr01^xxii^	85.031 (16)
B02^xi^—Cr02—Cr01^i^	124.601 (13)	B02^xiv^—B02—Cr01^xxiii^	65.376 (19)
B02^i^—Cr02—Cr01^i^	55.399 (13)	Cr01^x^—B02—Cr01^xxiii^	85.031 (16)
B02^xii^—Cr02—Cr01^i^	125.194 (13)	Cr01^xxi^—B02—Cr01^xxiii^	130.75 (4)
B02^xiii^—Cr02—Cr01^i^	54.806 (13)	Cr01^xxii^—B02—Cr01^xxiii^	74.894 (14)
Cr01^xi^—Cr02—Cr01^i^	180.0	B02^xiv^—B02—Cr02^iv^	117.946 (18)
B02^xi^—Cr02—Cr01^vii^	125.194 (13)	Cr01^x^—B02—Cr02^iv^	137.087 (3)
B02^i^—Cr02—Cr01^vii^	54.806 (13)	Cr01^xxi^—B02—Cr02^iv^	70.010 (2)
B02^xii^—Cr02—Cr01^vii^	55.399 (13)	Cr01^xxii^—B02—Cr02^iv^	70.010 (2)
B02^xiii^—Cr02—Cr01^vii^	124.601 (13)	Cr01^xxiii^—B02—Cr02^iv^	137.087 (3)
Cr01^xi^—Cr02—Cr01^vii^	110.203 (2)	B02^xiv^—B02—Cr02^vi^	117.946 (18)
Cr01^i^—Cr02—Cr01^vii^	69.797 (2)	Cr01^x^—B02—Cr02^vi^	70.010 (2)
B02^xi^—Cr02—Cr01^xv^	54.806 (13)	Cr01^xxi^—B02—Cr02^vi^	137.087 (3)
B02^i^—Cr02—Cr01^xv^	125.194 (13)	Cr01^xxii^—B02—Cr02^vi^	137.087 (3)
B02^xii^—Cr02—Cr01^xv^	124.601 (13)	Cr01^xxiii^—B02—Cr02^vi^	70.010 (2)
B02^xiii^—Cr02—Cr01^xv^	55.399 (13)	Cr02^iv^—B02—Cr02^vi^	124.11 (4)
Cr01^xi^—Cr02—Cr01^xv^	69.797 (2)	B02^xiv^—B02—Cr01	137.989 (14)
Cr01^i^—Cr02—Cr01^xv^	110.203 (2)	Cr01^x^—B02—Cr01	81.796 (6)
Cr01^vii^—Cr02—Cr01^xv^	180.0	Cr01^xxi^—B02—Cr01	81.796 (6)
B02^xi^—Cr02—Cr01^xiii^	54.806 (13)	Cr01^xxii^—B02—Cr01	139.630 (18)
B02^i^—Cr02—Cr01^xiii^	125.194 (13)	Cr01^xxiii^—B02—Cr01	139.630 (18)
B02^xii^—Cr02—Cr01^xiii^	124.601 (13)	Cr02^iv^—B02—Cr01	69.622 (17)
B02^xiii^—Cr02—Cr01^xiii^	55.399 (13)	Cr02^vi^—B02—Cr01	69.622 (17)
Cr01^xi^—Cr02—Cr01^xiii^	110.203 (2)	B02^xiv^—B02—Cr01^xxiv^	137.989 (14)
Cr01^i^—Cr02—Cr01^xiii^	69.797 (2)	Cr01^x^—B02—Cr01^xxiv^	139.630 (18)
Cr01^vii^—Cr02—Cr01^xiii^	108.017 (3)	Cr01^xxi^—B02—Cr01^xxiv^	139.630 (18)
Cr01^xv^—Cr02—Cr01^xiii^	71.983 (3)	Cr01^xxii^—B02—Cr01^xxiv^	81.796 (6)
B02^xi^—Cr02—Cr01^xvi^	124.601 (13)	Cr01^xxiii^—B02—Cr01^xxiv^	81.796 (6)
B02^i^—Cr02—Cr01^xvi^	55.399 (13)	Cr02^iv^—B02—Cr01^xxiv^	69.622 (17)
B02^xii^—Cr02—Cr01^xvi^	125.194 (13)	Cr02^vi^—B02—Cr01^xxiv^	69.622 (17)
B02^xiii^—Cr02—Cr01^xvi^	54.806 (13)	Cr01—B02—Cr01^xxiv^	84.02 (3)
Cr01^xi^—Cr02—Cr01^xvi^	108.017 (3)		

Symmetry codes: (i) −*y*+1, *x*, *z*; (ii) *y*−1, −*x*+1, −*z*; (iii) −*x*+1/2, −*y*+1/2, −*z*+1/2; (iv) *x*, *y*+1, *z*; (v) −*x*+1/2, −*y*+3/2, −*z*+1/2; (vi) −*x*+1/2, *y*+1/2, −*z*; (vii) −*x*, −*y*+1, *z*; (viii) *y*−1/2, −*x*+1/2, −*z*+1/2; (ix) −*y*+1/2, *x*+1/2, −*z*+1/2; (x) *y*, −*x*+1, *z*; (xi) *y*−1, −*x*, −*z*; (xii) −*x*, −*y*+1, −*z*; (xiii) *x*, *y*−1, *z*; (xiv) −*x*+1, −*y*+2, −*z*; (xv) *x*, *y*−1, −*z*; (xvi) −*y*+1, *x*, −*z*; (xvii) *y*−1, −*x*, *z*; (xviii) −*y*+1/2, *x*−1/2, −*z*+1/2; (xix) *x*−1/2, *y*−1/2, −*z*+1/2; (xx) −*x*, *y*, −*z*+1/2; (xxi) −*y*+1, *x*+1, *z*; (xxii) −*y*+1, *x*+1, −*z*; (xxiii) *y*, −*x*+1, −*z*; (xxiv) *x*, *y*, −*z*.
